# The Association between ABO and Rh Blood Groups and Risk of Endometriosis in Iranian Women

**DOI:** 10.22074/ijfs.2018.5435

**Published:** 2018-06-20

**Authors:** Farideh Malekzadeh, Ashraf Moini, Elham Amirchaghmaghi, Leila Daliri, Mohammad Reza Akhoond, Mehrak Talebi, Rihaneh Hosseini

**Affiliations:** 1 Department of Epidemiology and Reproductive Health, Reproductive Epidemiology Research Center, Royan Institute for Reproductive Biomedicine, ACECR, Tehran, Iran; 2 Department of Endocrinology and Female Infertility, Reproductive Biomedicine Research Center, Royan Institute for Reproductive Biomedicine, ACECR, Tehran, Iran; 3 Department of Gynaecology and Obstetrics, Arash Women’s Hospital, Tehran University of Medical Sciences, Tehran, Iran; 4 Vali-e-Asr Reproductive Health Research Center, Tehran University of Medical Sciences, Tehran, Iran; 5 Department of Regenerative Biomedicine, Cell Science Research Center, Royan Institute for Stem Cell Biology and Technology, ACECR, Tehran, Iran; 6 Department of Statistics, Mathematical Sciences and Computer Faculty, Shahid Chamran University of Ahvaz, Ahvaz, Iran

**Keywords:** ABO and Rh Blood Groups, Endometriosis, Women

## Abstract

**Background:**

Endometriosis is a common gynaecological disease that affects quality of life for women. Several studies have revealed that both environmental and genetic factors contribute to the development of endometriosis. The aim of this study was to investigate the distribution of ABO and Rh blood groups in Iranian women with endometriosis who presented to two referral infertility centers in Tehran, Iran.

**Materials and Methods:**

In this case-control study, women who referred to Royan Institute and Arash Women’s Hospital for diagnostic laparoscopy between 2013 and 2014 were assessed. Based on the laparoscopy findings, we categorized the women into two groups: endometriosis and control (women without endometriosis and normal pelvis). Chi-square and logistic regression tests were used for data analysis.

**Results:**

In this study, we assessed 433 women, of which 213 patients were assigned to the endometriosis group while the remaining 220 subjects comprised the control group. The most frequent ABO blood group was O (40.6%). The least frequent blood group was AB (4.8%). In terms of Rh blood group, Rh+ (90.1%) was more frequent than Rh- (9.9%). There was no significant correlation between ABO (P=0.091) and Rh (P=0.55) blood groups and risk of endometriosis. Also, there was no significant difference between the two groups with regards to the stage of endometriosis and distribution of ABO and Rh blood groups (P>0.05).

**Conclusion:**

Although the O blood group was less dominant in Iranian women with endometriosis, we observed no significant correlation between the risk of endometriosis and the ABO and Rh blood groups. Endometriosis severity was not correlated to any of these blood groups.

## Introduction

Endometriosis is a chronic gynaecological disorderwhich severely impacts the quality of life of affectedwomen (1). Endometriosis is defined as the presenceof endometrial tissue outside the uterine cavity. Thefrequency of endometriosis in reproductive age wom. en is 5-10%, whereas for infertile women it is 50% (2). This disease can lead to severe pain, inability toperform daily activities, and negatively affect familyrelationships, confidence, social functioning, sexual and mental health. The pathogenesis and mechanismsof endometriosis, which lead to the immigration of en. dometrial cells to areas outside the uterine cavity, hasnot been determined thus far. Several studies have sug. gested that endometriosis is caused by the interactionof genetic and environmental factors (2-4).

Identifying risk factors, especially in the severe form of endometriosis, can shorten the time between onset of symptoms and diagnosis. Proposed risk factors include low body mass index (BMI), family history or personal history of endometriosis, and dysmenorrhea (5-7). Moini et al. (8) have reported that age, BMI, duration of the menstrual cycle, history of abortion, dyspareunia, pelvic pain, and a family history of endometriosis are independent risk predictors for each type of endometriosis. 

On the other hand, several studies have shown a correlation between ABO and Rh blood groups with infectious and noninfectious diseases. In addition, the distribution of ABO and Rh blood groups varies worldwide because of geographical and ethnic differences (9-13). 

A biological explanation for the correlation between main blood groups and certain diseases has not been stated. Genomic studies have shown that single variations at the ABO locus and ABO blood groups were associated with pro-inflammatory cytokine tumour necrosis factor (TNF) receptor levels and adhesion molecules such as intercellular adhesion molecule (ICAM) and E- selectin (14-16). ABO and Rh blood groups have been reported as genetic risk factors for tumours and tumour- like diseases (17). The reason for this relationship remains unclear; however, an important assumption is that the ABO and Rh blood group antigens on the tumour cells facilitate tumour cell movement. Although several studies have assessed the relationship between ABO and Rh blood groups with endometriosis, their results were controversial (18-21).

The aim of present study was to identify any association between ABO and Rh blood groups with endometriosis. Identification of an association between ABO and Rh blood groups with endometriosis could assist with diagnosis at the early stages of this disease in at- risk women.

## Materials and Methods

In this case-control study, we assessed women who underwent diagnostic laparoscopy secondary to unexplained infertility or tubal ligation at Royan Institute and Arash Women’s Hospital between 2013 and 2014. The Ethics Committee of Royan Institute approved this study and all study participants signed an informed consent form. Inclusion criteria consisted of women with endometriosis or normal pelvis according to laparoscopic findings. Endometriosis was confirmed according to ASRM classification based on the features of the lesions during laparoscopy and pathology reports. A total of 433 women were assigned to the endometriosis and control (women without endometriosis and normal pelvis) groups. In the endometriosis group, we determined the stage of endometriosis according to ASRM classification (22) based on type and distribution of lesions, adhesion or existence of endometrioma.

ABO and Rh blood group typing were performed before laparoscopy at laboratories located in Royan Institute and Arash Women’s Hospital. A questionnaire was completed for each woman. 

### Statistical analysis

Statistical analysis was performed by using the Statistical Package for the Social Sciences (SPSS) version 20. The chi-square, t test, one-way ANOVA, and logistic regression tests were used for data analysis. Ordinal logistic regression was used to examine the relationship between the ABO and Rh blood groups to stages of endometriosis. Data are presented as number (percent) or mean ± SD. P<0.05 were considered significant.

## Results

In this study there were 433 participants. According to laparoscopy findings, 213 women with endometriosis were placed in the endometriosis group and 220 subjects with normal pelvis comprised the control group. 

The 433 enrolled women had a mean age of 30.74 ± 5.97 years. The mean age of the endometriosis groups was 30.43 ± 5.81 years and the control group was 31.05 ± 6.11 years, which was not statistically significant [P=0.27, odds ratio (OR): 0.98, 95% confidence interval (CI): 0.95-1.01]. 

The average BMI in the endometriosis group was 23.48 ± 3.38 kg/m^2^. The BMI of the control group was 25.88 ± 4.40 kg/m^2^. Statistical analysis showed a significant inverse association between BMI and endometriosis (P<0.001, OR: 0.85, 95% CI: 0.80-0.90). The OR indicated that each unit increase in BMI decreased the risk of endometriosis. 

In this study, the most common ABO blood group was O with a frequency of 40.6%. There was no significant association between ABO blood groups and endometriosis (P=0.091), although the risk of endometriosis in the O blood group was lower than the other blood groups. The Rh+ blood group was present in 390 women (90.1%). There was no significant correlation between Rh blood group and risk of endometriosis (P=0.55, Table 1).

Among demographic variables, regression analysis indicated that a positive family history of endometriosis in first-degree relatives had a significant association with the risk of endometriosis (P=0.004, OR: 8.85, 95% CI: 2.01-38.98). The risk of endometriosis in women whom first-degree relatives (mother, sister) suffered from endometriosis was approximately 9 times more than women without a positive family history (Table 2).

In this study, the most common symptoms of endometriosis were pain during menstruation (73.7%) and infertility (56.3%). There was no significant association between the different stages of endometriosis (I-IV) and frequency of ABO and Rh blood groups (Table 3).

One-way ANOVA was used to compare mean age and BMI of women in different stages of endometriosis. Mean age and BMI did not significantly differ in the different stages of endometriosis (Table 4).

**Table 1:** The frequency of ABO and Rh blood groups in the endometriosis and control groups


Blood groups	Endometriosis groupn (%)	Control groupn (%)	OR (95% CI)	P value

A	73 (34.3)	73 (33.2)	1.316 (0.84-2.04)	0.091
B	50 (23.5)	40 (18.2)	1.645 (0.98-2.74)	
AB	14 (6.5)	7 (3.2)	2.63 (1.01-6.83)	
O	76 (35.7)	100 (45.4)	Reference level	
Rh+	190 (89.2)	200 (90.9)	Reference level	0.55
Rh-	23 (10.8)	20 (9.1)	1.21 (0.64-2.27)	


OR; Odds ratio and CI; Confidence interval.

**Table 2:** Relation between history of endometriosis in first-degree relatives with the risk of endometriosis


Family history of endometriosis	Endometriosis groupn (%)	Control groupn (%)	OR (95% CI)	P value

No	197 (92.5)	218 (99.1)	Reference level	0.004
Yes	16 (7.5)	2 (0.9)	8.85 (2.01-38.98)	


OR; Odds ratio and CI; Confidence interval.

**Table 3:** Association of severity of endometriosis with ABO and Rh blood groups


Blood group	Control groupn=220	Endometriosis groupn=213				P value^c^	P value^b^	OR (95% CI)^a^
		Stage 1	Stage 2	Stage 3	Stage 4			

A	73 (33.2)	6 (27.3)	7 (30.4)	27 (36.5)	33 (35.1)	0.053	---	Reference level
B	40 (18.2)	4 (18.2)	2 (8.7)	19 (25.7)	25 (26.6)		0.292	1.297 (0.799-2.106)
AB	7 (3.2)	3 (13.6)	3 (13)	1 (1.4)	7 (7.4)		0.310	1.542 (0.668-3.557)
O	100 (45.4)	9 (40.9)	11 (47.8)	27 (36.5)	29 (30.9)		0.127	0.723 (0.477-1.096)
Rh+	200 (90.9)	20 (90.9)	20 (87)	65 (87.8)	85 (90.4)	0.871	---	Reference level
Rh-	20 (9.1)	2 (9.1)	3 (13)	9 (12.2)	9 (9.6)		0.680	1.130 (0.630-2.031)


Values are given as number (%). ^a^; Results are reported based on the odds ratio (OR) obtained from ordinal logistic regression, ^b^; P value based on ordinal logistic regression, and ^c^; P value based on Fisher’s exact test.

**Table 4:** The frequency of different stages of endometriosis, age, and body mass index (BMI) in the endometriosis group


Stage of endometriosis	n (%)	Age (Y) Mean ± SD	BMI (kg/m^2^)Mean ± SD

Stage 1	22 (10.3)	31.59 ± 4.53	23.65 ± 3.38
Stage 2	23 (10.8)	29.83 ± 4.98	23.72 ± 2.78
Stage 3	74 (34.8)	30.64 ± 5.89	23.17 ± 3.61
Stage 4	94 (44.1)	30.14 ± 6.22	23.62 ± 3.36
Total	213 (100)	30.43 ± 5.81	23.48 ± 3.38
P value -		0.696	0.812


## Discussion

Several studies have shown that the distribution of Rh and ABO blood groups can depend on ethnicity and change in a geographic area over time (23-28). In the present study, we have determined that O was the most common ABO blood group and the least frequent group was AB. The most common Rh blood group was Rh+. These findings agreed with studies in Iran conducted by Pourfathollah et al. (29) and Hosseini et al. (30). 

Although the result of our study showed that the risk of endometriosis in women with blood group O was lower than other ABO blood groups, this difference was not statistically significant. The risk of endometriosis in all ABO blood groups was similar, which was consistent with studies by Daliri et al. (31) in Fars Province, Southern Iran, Kim and his colleague (32) in Korea, and Borghese et al. (33) in France. However, these results differed from findings reported by Demir et al. (21) in Turkey and Matalliotakis et al. (20). They reported that the highest risk of endometriosis was among women with blood group A and the lowest risk was seen in women with blood group O.

In the present study, the most frequent Rh blood group was Rh+. There was no significant relationship observed between Rh and the risk of endometriosis, which was consistent with another study in Iran by Daliri et al. (31). However, Borghese et al. (33) in France reported that the chance of developing endometriosis was double in Rh- women and Demir et al. (21) reported that the risk of endometriosis in Rh+ women was significantly higher than the control group.

The results of our study showed that the different stages of endometriosis and ABO and Rh blood groups did not have a significant relationship, which was consistent with findings by Demir et al. (21) and Matalliotakis et al. (20).

 The limitations of this study included difficulties in finding women with normal pelvis during laparoscopy and low sample size based on the study power of 0.56. The power of this study indicated the need to conduct a study with a larger sample size.

## Conclusion

This study has shown no significant association between the main blood groups (ABO and Rh) and the risk of endometriosis. It seems that Iranian women with the O blood group are less likely to develop endometriosis compared to women with other blood groups. However, this finding should be confirmed with a larger study population. In addition, the severity of endometriosis was not correlated to any of these blood groups. 
